# Inhibition realization of multidrug resistant bacterial and fungal isolates using *Coccinia indica* extracts

**DOI:** 10.1016/j.sjbs.2022.01.045

**Published:** 2022-01-24

**Authors:** Mohammad Y. Alshahrani, Essam H. Ibrahim, Mohammed Asiri, Mona Kilany, Ahmad Alshehri, Ali G. Alkhathami, Kareem Morsy, Harish C. Chandramoorthy

**Affiliations:** a Department of Clinical Laboratory Sciences, College of Applied Medical Sciences, King Khalid University, P.O. Box 61413, Abha 9088, Saudi Arabia; b Research Center for Advanced Materials Science (RCAMS), King Khalid University, P.O. Box 9004, Abha 61413, Saudi Arabia; c Biology Department, Faculty of Science, King Khalid University, P.O. Box 9004, Abha 61413, Saudi Arabia; d Blood Products Quality Control and Research Department, National Organization for Research and Control of Biologicals, Cairo 12611, Egypt; e Department of Microbiology, National Organization for Drug Control and Research (NODCAR), Cairo 12561, Egypt; f Department of Clinical Laboratory Sciences, College of Applied Medical Sciences, Najran University, P.O. Box 1988, Najran, Saudi Arabia; g Department of Zoology, Faculty of Science, Cairo University, 12613 Cairo, Egypt; h Department of Microbiology and Clinical Parasitology, College of Medicine, King Khalid University, P.O. Box 9004, Abha 61413, Saudi Arabia

**Keywords:** Antibacterial, Antifungal, *Coccinia indica*, Multi drug resistant bacteria, Multi drug resistant fungi, Methicillin resistant *Staphylococcus aureus*

## Abstract

The crude aqueous and ethanolic leaf extracts of *Coccinia indica* were screened for methicillin resistant *Staphylococcus aureus* (MRSA)*,* multidrug resistant (MDR) *Streptococcus pyogenes*, *Escherichia coli*, *Candida auris* and *Trichophyton rubrum*. Antibacterial and antifungal activities were assessed by standard disc diffusion and tube dilution methods. The results showed that ethanolic extract inhibited *MRSA*, *C. auris* at 250 µg/mL and *S. pyogenes* at 200 µg/mL comparable to the susceptible antibiotics used as positive controls. There was no observable activity against *T. rubrum*, while a mild activity was observed with ethanolic extracts over *E. coli* at higher concentrations which did not turn out to be complete or significant inhibition. Aqueous extract did not exhibit any observable activity over the five organisms tested. Furthermore, the results showed clear cut concentration dependent antibacterial and antifungal activities with additional variation of specific activity over Gram positive and negative bacteria, yeast and filamentous fungi. So, it is evident that ethanolic extract of *Coccinia indica* could be further escalating for mechanistic studies in the era of multidrug resistance, indigenous preparations from herbs could be a safe choice over clinically challenging organisms.

## 1 Introduction

Multidrug resistance is a huge problem across globe irrespective of developed or developing world (Aslam et al., 2018). Currently drug resistance forms the basis of the pharmaceutical research especially in the area of drug discovery (Simpkin et al., 2017). Antibiotic and antifungal drug resistant organisms and search for proper drugs to combat them have been always prioritized in biomedical research. Many lead molecules either form natural products, herbs, herbal preparations, small molecules form synthetic and semi synthetic libraries (Atanasov et al., 2021). For past three decades herbs and herbal products used in the traditional system of medicine have been widely researched for their immense pharmacological and biological activities (Yuan et al., 2016). It may be noted that, traditional preparations have been usually done with help of water (aqueous) or sometimes with alcohol (ethanol). Most of the herbs or plant extracts have been used to treat specific systemic diseases or symptoms, while some of them as general ingredient in all the medications with or without knowing their exact mechanism of action (Ekor, 2014). In recent times there are many herbal drugs to treat microbial infections and surprisingly herbs and plants of the same family and different subspecies vary in their antimicrobial activity (Gutiérrez et al., 2018). Recent technological advancement and research has provided a gate way for scientifically authenticate these herbs and preparations against multi drug organisms. Though there are many reports on the ingredients of the plant or part of the plant used, we have commonly accepted the formula of traditional formulations in modern medical applications.

In the current investigation, a fast-growing tropical vine *Coccinia indica* also known as ivy gourd or baby watermelon was selected on the basis of its profound biological and pharmacological activities observed in the literature (Niazi et al., 2009, Raje et al., 2013). Though there some studies pertaining to its antibacterial (Devi et al., 2021) and antifungal activities however, with different species (Venkateswaran and Pari, 2003) almost all parts of the plants have been used as drug in traditional medical branches of Ayurveda and Unani. Many diseases like leprosy, tumor and jaundice have been treated with extract of fruits and leaves due to its antioxidant nature while, animal studies have shown to reduce plasma glucose levels in diabetes (Shifali et al., 2021). Further, dried flower and leaf of the plant is used to treat variety of clinical conditions like eye irritations, skin eruptions, burns, nausea and earache (Ramachandran et al., 2014). There are a few experimental works to demonstrate the antiviral and anti-helminthic activity (Arbab et al., 2017). The insulin stimulatory effect and antioxidant properties of the leaf extract was proved recently (Mukherjee et al., 1972).

Therefore, with all these available background studies, it was decided to assess the antibacterial and antifungal activity of the leaf extracts against MDR clinical isolates. To add the reason behind use of the whole extract was authentication of complete classical extracts used in the traditional medical practices.

## 2 Materials and methods

### 2.1 Preparation of aqueous and ethanolic extracts

Fresh leaves of *Coccinia indica* (Cucurbitaceae) were collected from plantations in the south western Saudi Arabia. The leaves were cleaned, dried in shade, crushed to get powder form using a blender (at low-speed) and stored in air tight opaque containers till further extracted. About 100 g of the dried powdered leaves were mixed with 100 mL water and another 100 g were mixed with 100 mL ethanol (absolute). The mixtures were kept individually in the rotary shaker (60 rpm) for 2 days. Separate mixtures were consequently filtered using muslin cloth followed by desiccation at 55 °C. The desiccated extracts were stored at 4 °C in air tight vials. Thereafter, 100 mg of the aqueous and ethanolic extracts were dissolved in 10 mL sterile distilled water and 10 mL of dimethyl formamide (DMFO, Sigma-Aldrich) respectively and filtered through 0.45 µm filter for the examination of microbial assay.

### 2.2 Bacterial and fungal cultures

The MRSA and MDR isolates of *Escherichia coli, Streptococcus pyogenes, Candida auris* and *Trichophyton rubrum* were obtained from our clinical isolate collection at Department of Microbiology, College of Applied Medical Sciences, King Khalid University, Abha, Saudi Arabia. The isolates were previously identified using recommended morphological and biochemical tests (data not shown). The bacterial cultures were maintained in Muller Hinton broth media (MHB) at 37 °C and preserved in nutrient agar slopes at 4 °C tell use. The fungal cultures were maintained at 28 °C in Sabouraud Dextrose Agar with Chloramphenicol 0.05% (SDA) and subsequently sub-cultured in potato dextrose agar (PDA) for inducing sporulation. Antibiotic sensitivity pattern was determined by Bauer et al. method (Bauer et al., 1966).

### 2.3 Disc diffusion method

The MRSA and MDR bacterial isolates were surface swabbed in Muller-Hinton agar (MHA) plates with 100 μL of the logarithmic phase bacteria and fungi at a concentration set to 0.5 McFarland turbidity standard (10^8^ cfu/mL). Previously prepared aqueous and ethanolic Coccinia extract saturated 0.7 cm discs were placed onto the plates with 2 cm space and the plates were kept at 37/30 °C for 28/48 h for bacterial and fungal antimicrobial potency, respectively. MIC was inferred as the least concentration of the extracts that prevented the visible growth “zone of clearance” of the bacterial cultures (Harish et al., 2010). For the sensitivity test of Candida, MHA with 2% glucose and 0.5 µg/mL methylene blue dye (MHA-GMB) was used. Where the yeast suspension was prepared using sterile 0.85% saline and the turbidity adjusted to 0.5 McFarland standards with concentration 1 × 10^6^ cells per mL. A semiconfluent lawn culture was made using sterile cotton swab and the extract saturated discs were placed.

### 2.4 Tube dilution method

Traditional tube dilution method was followed for antibacterial and antifungal screening. Serial dilutions of each extract from 10 mg/mL to 1 mg/mL were prepared in 2.6 mL MHB reaching a final volume of 3.6 mL. Initial OD was measured at 590 nm, to which 0.45 mL of the bacterial suspension (1X10^6^ cfu/mL) was added to make the final volume as 4 mL. Post incubation of tubes at 37 °C (bacterial) for 24–48 h and 30 °C (fungal) for 48–96 h, then the final OD was measured. The difference between the initial OD and final OD determined the inhibition potency of bacterial growth. Blank tubes containing sterile media and culture tubes containing 0.4 mL (1 × 10^6^ cfu/mL) of the bacterial or fungal suspension served as negative and positive control respectively. The MIC was calculated as the extract concentration showing fall in the OD compared to positive control (Helal et al., 2019, Manavathu et al., 1996).

### 2.5 Statistical analysis

All the experiments were done in triplicate unless specified. Statistics were done with Graph Pad Prism v.6.0. Statistical significance was assessed through one way ANOVA and significances were denoted by p < 0.05. All data were presented as mean values ± standard error (SE).

## 3 Results

Results depicted in Table 1, Table 2 confirmed the multi-drug resistance as well as the antibiotic/fungal sensitivity pattern of the clinical isolates. From the current investigation it is evident that ethanolic extract of *Coccinia indica* exhibited antibacterial and antifungal bioactivity in significant manner than the aqueous extract (Table 3). Since ethanol was used for extraction, the residual traces of ethanol in the dried extracts were tested with routine alcohol identification test that gave negative results.

**Table 1:** Antibiotic susceptibility pattern of the clinical isolates.

Antibiotics	Clinical Isolates		
	*MRSA*	*S. pyogenes*	*E. coli*
Ampicillin (A)	R	R	R
Amoxiclav (Ac)	R	R	R
Amikacin (Ak)	S	NA	R
Ceftazidime (Ca)	R	NA	R
Cefotaxime (Ce)	R	S	R
Ciprofloxacin (Cf)	R	NA	R
Cefuroxime (Cu)	R	S	R
Cefazolin (Cz)	R	S	R
Gentamicin (G)	S	NA	R
Imipenem (I)	**NA**	NA	I
Nalidixic acid (Na)	**NA**	NA	R
Nitrofurantoin (Nf)	S	NA	S
Norfloxacin (Nx)	R	NA	R
Erythromycin(E)	**R**	S	**NA**
Clindamycin (Cd)	**S**	S	**NA**
Penicillin (P)	R	S	**NA**
Rifampicin (R)	R	**NA**	**NA**
Vancomycin (Va) E test	**S**	**NA**	**NA**

Where R: resistant; S: susceptible and NA: not applicable.

**Table 2:** Antifungal susceptibility pattern of the clinical isolate.

Antifungal drugs	Clinical Isolates	
	*C.auris*	*T.rubrum*
Fluconazole -F	R	S
Voriconazole -V	R	S
Anidulafungin-Ani	S	S
Amphotericin B- Amp	S	S

Where (R) stands for resistant, (S) stands for susceptible and (NA) stands for not applicable.

**Table 3:** Summary of antibacterial and fungal activity by both disc diffusion and tube dilution technique.

Extracts	Concentration of the extracts in μg/mL	Clinical Isolates									
		*MRSA*		*S.pyogenes*		*E.coli*		*C.auris*		*T.rubrum*	
		*Disc*	*Tube*	*Disc*	*Tube*	*Disc*	*Tube*	*Disc*	*Tube*	*Disc*	*Tube*
Aqueous *C. indica*	25	NDA	NDA	NDA	NDA	NDA	NDA	NDA	ND	ND	NDA
	50	NDA	NDA	NDA	NDA	NDA	NDA	NDA	ND	ND	I
	100	NDA	NDA	I	I	NDA	NDA	NDA	ND	ND	I
	200	NDA	NDA	I	I	NDA	NDA	NDA	ND	ND	I
	250	NDA	NDA	I	I	NDA	NDA	NDA	ND	ND	I
	300	NDA	NDA	I	I	NDA	NDA	NDA	ND	ND	I

Ethanolic *C. indica*	25	I	I	I	I	NDA	NDA	I	ND	ND	I
	50	I	I	M	M	NDA	NDA	I	ND	ND	I
	100	M	M	S	S	I	I	I	ND	ND	I
	200	S	S	HS	HS	I	I	M	ND	ND	I
	250	HS	HS	HS	HS	M	M	HS	ND	ND	I
	300	HS	HS	HS	HS	M	M	HS	ND	ND	I

Summary of antibacterial and fungal activity by both disc diffusion and tube dilution technique. Where ND: not done; NDA: no detectable activity; I: intermediate (Very marginal reduction of OD values); M: mild (Considerable reduction in OD values due to slow growth/altered growth pattern); S: sensitive (Above 70% reduction in the OD values) and HS: highly sensitive (No detectable growth of organisms).

**Fig. 1:**
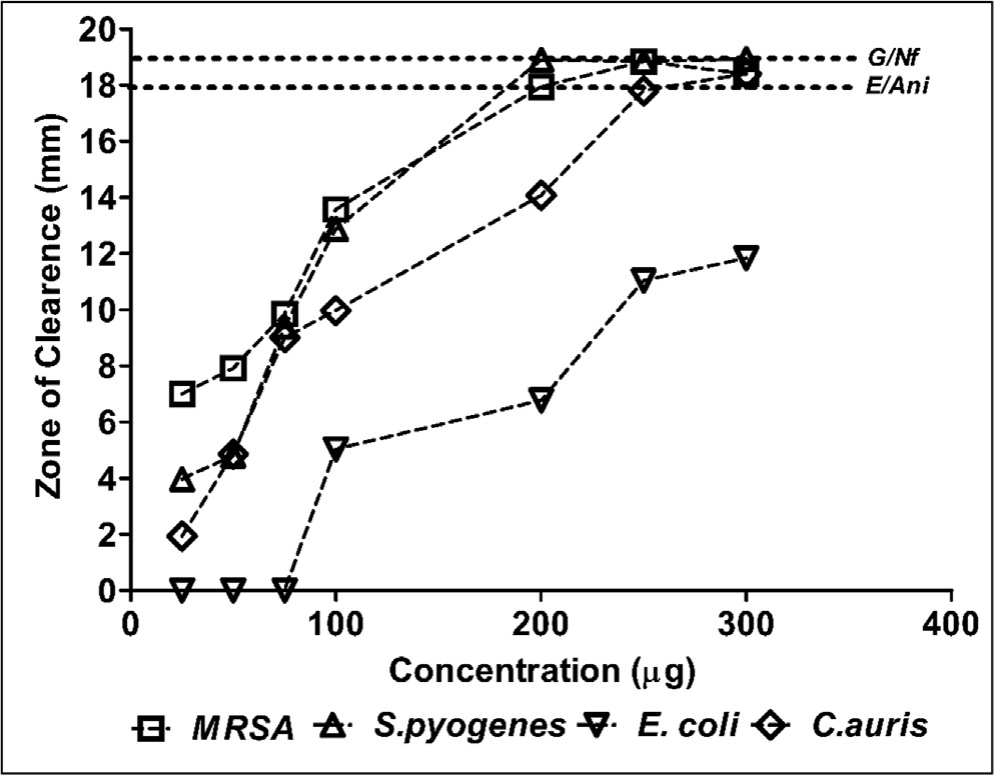
Antibacterial and fungal activity of ethanolic extract of *Coccina indica* by disc diffusion method. Zone of clearance is measured in mm and compared to the susceptible control antibiotics and antifungal drug.

The results of the disc diffusion method showed that Coccinia ethanolic extract exhibited comparable activity against MRSA at 250 μg, *S. pyogenes* at 200 μg and *C. auris* at 250 μg with its antibiotic and antifungal controls (Fig. 1 & Table 4, Table 5).

**Table 4:** Antibacterial and fungal activity by disc diffusion method.

Herb	Nature of extract	Clinical Isolates	Concentration (μg/mL) of the extract versus Zone of Clearance (mm)*						
			25	50	75	100	200	250	300
*Coccinia indica*	Aqueous	*MRSA*	**-/-**	**-/-**	**-/-**	**-/-**	**-/-**	**-/-**	**-/-**
		*S.pyogenes*	**-/-**	**-/-**	**-/-**	4	4	**4**	5
		*E.coli*	**-/-**	**-/-**	**-/-**	**-/-**	**-/-**	**-/-**	**-/-**
		*C.auris*	**-/-**	**-/-**	**-/-**	**-/-**	**-/-**	**-/-**	**-/-**
	Ethanolic	*MRSA*	**7**	**8**	**10**	**14**	**18**	**19**	**18**
		*S.pyogenes*	**4**	**5**	**10**	**13**	19	19	19
		*E.coli*	**-/-**	**-/-**	**-/-**	**5**	**7**	**11**	**12**
		*C.auris*	2	5	9	10	14	18	18

*The results are expressed as average zone of inhibition (mm) from three independent experiments. Where (-/-) stands for Nil clearance.

**Table 5:** Reference sensitivity of positive controls used along with the disc diffusion test.

Antibiotics	Zone of Clearance (mm)*			
	*MRSA*	*S.pyogenes*	*E.coli*	*C.auris*
**Gentamicin (G)**	**19**	**NA**	**NA**	**NA**
**Erythromycin(E)**	**NA**	**19**	**NA**	NA
**Nitrofurantoin (Nf)**	**NA**	**NA**	**18**	**NA**
**Anidulafungin (Ani)**	**NA**	**NA**	**NA**	**18**

Where NA: not applicable.

**Fig. 2:**
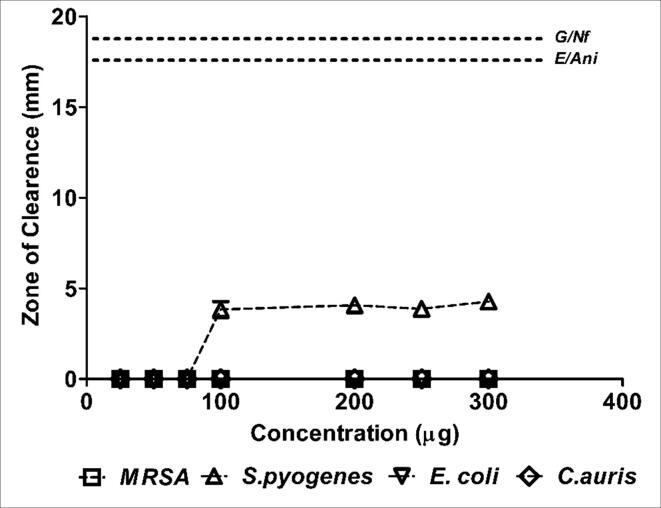
Antibacterial and fungal activity of aqueous extract of *Coccina indica* by disc diffusion method. Zone of clearance is measured in mm and compared to the susceptible control antibiotics and antifungal drug.

Indeed, concentration dependent activity against *MRSA*, *S. pyogenes* and *C. auris* were evident (Fig. 1 & Table 4, Table 5) showing a direct relationship with the active ingredients of the extract. Additionally, it may be further noted that the ethanolic extract exhibited a mild activity against *E.coli* (Fig. 1 & Table 4, Table 5) however it failed to exhibit fullest activity comparable to antibiotics used as positive control even at higher concentration (Data not shown). The Coccinia aqueous extract exhibited very low bioactivity against *S. pyogenes* (Fig. 2 & Table 4, Table 5).

**Fig. 3:**
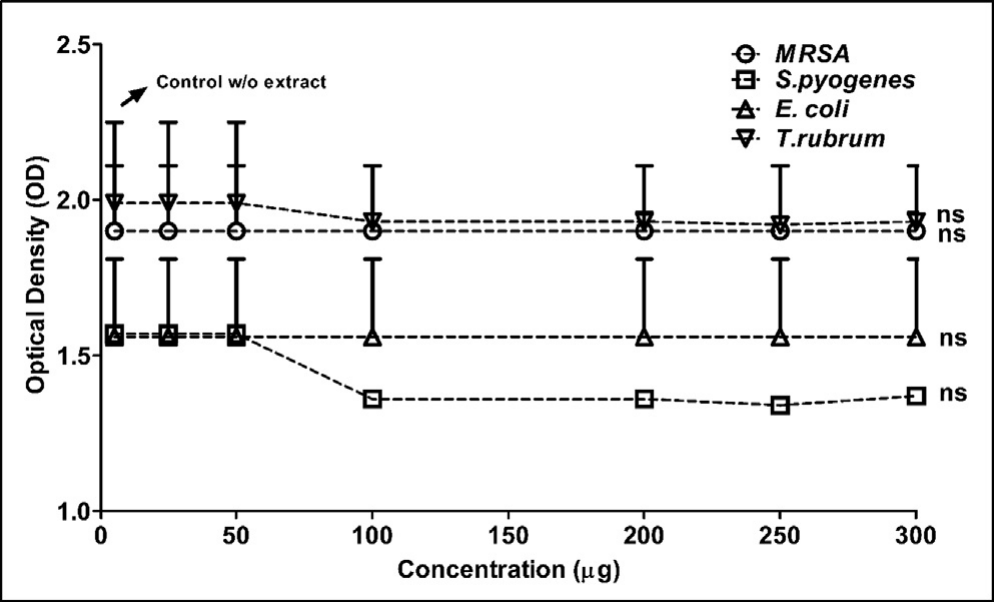
Antibacterial and fungal activity of aqueous extract of *Coccina indica* by tube dilution method. Reduction in the optical density (OD) of the extract added tubes are compared to the positive control (tube with only organism).

**Fig. 4:**
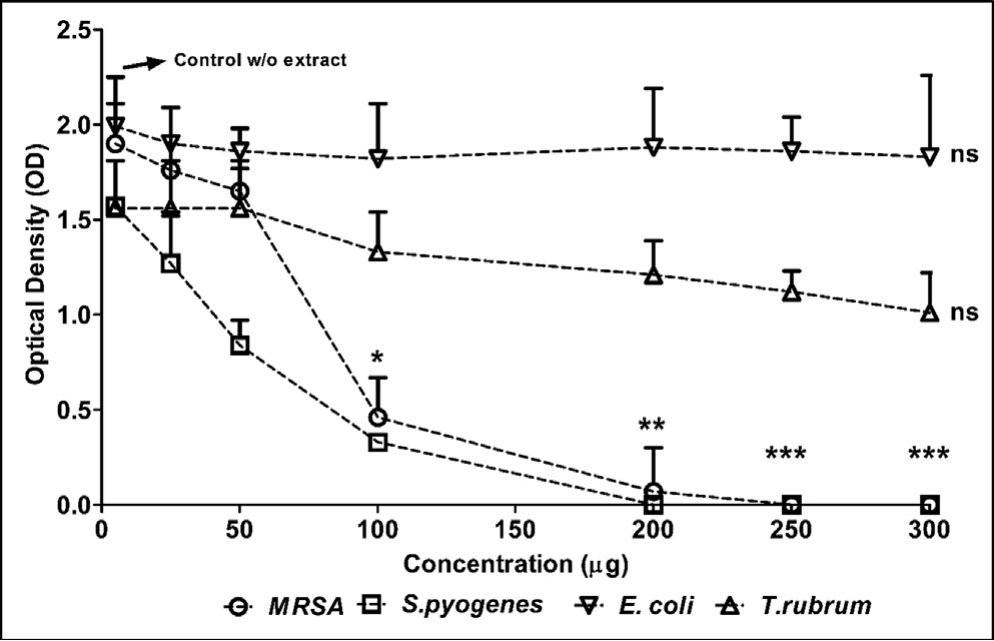
Antibacterial and fungal activity of aqueous extract of *Coccina indica* by tube dilution method. Reduction in the optical density (OD) of the extract added tubes are compared to the positive control (tube with only organism).

Concomitantly, the results of the tube dilution method augmented the results of disc diffusion method well (Fig. 3, Fig. 4 & Table 6).

**Table 6:** Antibacterial and fungal activity using tube dilution method.

Herb	Nature of extract	Clinical Isolates	Positive control OD (nm) Mean ± SE	Concentration (μg/mL) of the extract versus growth OD (nm) values expressed **as** Mean ± SE					
				25	50	100	200	250	300
*Coccinia indica*	Aqueous	*MRSA*	1.90 ± 0.21	NDA	NDA	NDA	NDA	NDA	NDA
		*S.pyogenes*	1.57 ± 0.24	NDA	NDA	1.36 ± 0.02	1.36 ± 0.01	1.34 ± 0.01	1.37 ± 0.01
		*E.coli*	1.56 ± 0.25	NDA	NDA	NDA	NDA	NDA	NDA
		*T.rubrum*	1.99 ± 0.26	NDA	NDA	1.93 ± 0.01	1.93 ± 0.03	1.92 ± 0.02	1.93 ± 0.03
	Ethanolic	*MRSA*	1.90 ± 0.21	1.76 ± 0.33	1.65 ± 0.12	0.46 ± 0. 21	0.07 ± 0.23	0	0
		*S.pyogenes*	1.57 ± 0.24	**1.27 ± 0.27**	**0.84 ± 0.13**	**0.33 ± 1.01**	**0**	0	0
		*E.coli*	1.56 ± 0.25	NDA	NDA	1.33 ± 0.21	1.21 ± 0.18	1.12 ± 0.11	1.01 ± 0.21
		*T.rubrum*	1.99 ± 0.26	1.90 ± 0.01	1.86 ± 0.12	1.82 ± 0.29	1.88 ± 0.31	1.86 ± 0.18	1.83 ± 0.43

The results are expressed as Mean ± SE of the OD values obtained compared to positive control without any susceptible antibiotics. Where NDA: no detectable activity; 0: no growth.nmm.

To add, the reduction of OD was not observed in the aqueous extract while the statistically significant OD reduction was observed at 100 μg for *MRSA* and *S. pyogenes* while *E. coli* and *T. rubrum* did not show any observable activity with both aqueous and ethanolic Coccinia extracts (Fig. 3, Fig. 4 & Table 6). Further clear dose dependent reduction of OD was observed with this method is validating the result of disc diffusion too. To add, we did not test *C. auris* by tube dilution and *T. rubrum* by disc diffusion as their growth pattern was slow inversely proportional to inoculum.

To summarize (Table 3) from both disc and tube dilution method the ethanolic extract of Coccinia exhibited profound activity against Gram +ve organisms and yeast like organisms while showed little activity against Gram −ve organisms and dermatophytes or filamentous fungi.

## 4 Discussion

The antibacterial and fungal effects of the ethanolic extracts of Coccinia leaf extracts showed promising activity against three out of five organisms tested. *MRSA, S. pyogens* and *C. auris* were susceptible to the ethanolic extract while aqueous extract did not have any observable activity on any of the tested organisms. *E. coli* and *T. rubrum* on the other hand were not susceptible to the both the extracts of Coccinia leaves. We used only crude extracts in this study as many of the previous literature and our observation has always turned to be in favor of crude over isolated compounds as later usually does not exhibit an observable property compared to the crude extract.

It is evident from current study and other studies elsewhere (Oboh et al., 2008, Yi et al., 2012) that, the ethanolic extracts usually have higher efficacy and activity compared to the aqueous extracts for the fact that, the former contains more secondary metabolites (Yi et al., 2012) extractable in ethanol and other higher polar solvents (Abubakar and Haque, 2020) than the aqueous extraction. Traditionally aqueous extracts of leaves served mostly as topical applications for bacterial infections (Cowan, 1999) and in advanced cases alcohol was used for extraction. The results of the ethanolic extract clearly indicated the presence of soluble metabolites resulting in dose dependent activity (Gonelimali et al., 2018). These observations well corroborated with the literature evidences preferring ethanolic extracts over aqueous extracts used at higher concentrations (Chassagne et al., 2020) and for longer periods (Silva et al., 2016) in treating infectious diseases.

The results of the antibacterial and fungal efficacy of Coccinia leaves were different from many traditional herbal studies. For instance, the ethanolic extract showed profound activity over gram +ve and yeast like organisms than gram -ve or filamentous fungi. Further this property may be attributed to the secondary metabolites exhibiting specific mechanism over gram +ve organisms (Gorlenko et al., 2020, Jakubiec-Krzesniak et al., 2018). These results were little varied with classical understanding of the antibacterial or antifungal studies (Bhalodia and Shukla, 2011). It may be noted that crude extracts usually posses’ mixture of chemical constituents which usually exhibits mixed activity when tested on broad range of microorganisms inclusive bacteria, viruses, fungi and parasites (Noorulla et al., 2009, Sakharkar and Chauhan, 2017). Though selective and specific activities over organisms are not common phenomenon observed with herbal extracts, it is evidenced form the observation that both aqueous and ethanolic extracts of Coccinia showed a feeble activity over *E.coli*. However, the results could be escalated to complete inhibition of *E.coli*. This may be due to less availability of the metabolite in the ethanol extract or loss of activity due to ethanol has to be further investigated with the use of higher polar solvents for the extraction.

It may be noted that some of important bioactivity of Coccinia fruits has been documented (Kondhare and Lade, 2017, Shaheen et al., 2011) over metabolic diseases and some studies have been undertaken in *Coccinia indica* and other subspecies over various microorganisms. However, emphasis over antibiotic resistant organisms of both bacteria and fungi have not been addressed with herbal extracts. Even with few of such studies, the results have not been escalated to higher trials nor authenticated to be used in the therapy. The ethanolic extract of Coccinia turned out be a good candidate to be further tested for antibiotic resistant gram +ve bacteria and yeast like fungi to deduce the mechanism of the action.

## 5 Conclusion

The results of the ethanolic extracts of *Coccinia indica* over all the five organisms by both disc diffusion and tube dilution methods were comparative and results were well in agreement with each other. The extracts were move active over gram +ve and yeast like organisms compared to gram −ve and filamentous fungi. The potential application of ethanolic extracts specially to treat gram +ve organisms and candida will be an addition to the list of lead molecules from the natural origin to combat drug resistance in common. To add, future analysis would be warranted to authenticate the bioactivity of this leaf extract in higher disease models.

## Declaration of Competing Interest

The authors declare that they have no known competing financial interests or personal relationships that could have appeared to influence the work reported in this paper.

## References

[b0005] Abubakar A.R., Haque M. (2020). Preparation of medicinal plants: basic extraction and fractionation procedures for experimental purposes. J. Pharm. Bioallied Sci..

[b0010] Arbab A.H., Parvez M.K., Al-Dosari M.S., Al-Rehaily A.J. (2017). In vitro evaluation of novel antiviral activities of 60 medicinal plants extracts against hepatitis B virus. Exp. Ther. Med..

[b0015] Aslam B., Wang W., Arshad M.I., Khurshid M., Muzammil S., Rasool M.H., Nisar M.A., Alvi R.F., Aslam M.A., Qamar M.U., Salamat M.K.F., Baloch Z. (2018). Antibiotic resistance: a rundown of a global crisis. Infect. Drug Resist..

[b0020] Atanasov A.G., Zotchev S.B., Dirsch V.M., Supuran C.T. (2021). Natural products in drug discovery: advances and opportunities. Nat. Rev. Drug Discov..

[b0025] Bauer A.W., Kirby W.M.M., Sherris J.C., Turck M. (1966). Antibiotic susceptibility testing by a standardized single disk method. Am. J. Clin. Pathol..

[b0030] Bhalodia N.R., Shukla V.J. (2011). Antibacterial and antifungal activities from leaf extracts of Cassia fistula l.: An ethnomedicinal plant. J. Adv. Pharm. Technol. Res..

[b0035] Chassagne F., Samarakoon T., Porras G., Lyles J.T., Dettweiler M., Marquez L., Salam A.M., Shabih S., Farrokhi D.R., Quave C.L. (2020). A systematic review of plants with antibacterial activities: A taxonomic and phylogenetic perspective. Front. Pharmacol..

[b0040] Cowan M.M. (1999). Plant products as antimicrobial agents. Clin. Microbiol. Rev..

[b0045] Devi R.S., Ganesh P.S., Girija A.S.S., Priyadharshini J.V. (2021). Attenuation of Quorum Sensing Controlled Virulence Factors and Biofilm Formation by Edible Fruit Extract of Coccinia indica against Pseudomonas aeruginosa. J. Pharm. Res. Int..

[b0050] Ekor M. (2014). The growing use of herbal medicines: issues relating to adverse reactions and challenges in monitoring safety. Front. Pharmacol..

[b0055] Gonelimali F.D., Lin J., Miao W., Xuan J., Charles F., Chen M., Hatab S.R. (2018). Antimicrobial properties and mechanism of action of some plant extracts against food pathogens and spoilage microorganisms. Front. Microbiol..

[b0060] Gorlenko, C.L., Kiselev, H.Y., Budanova, E. V, Zamyatnin, A.A.J., Ikryannikova, L.N., 2020. Plant Secondary Metabolites in the Battle of Drugs and Drug-Resistant Bacteria: New Heroes or Worse Clones of Antibiotics? Antibiot. (Basel, Switzerland) 9. 10.3390/antibiotics9040170 10.3390/antibiotics9040170 PMC7235868 32290036

[b0065] Gutiérrez, Y.I., Scull, R., Monzote, L., Rodríguez, K.M., Bello, A., Setzer, W.N., 2018. Comparative Pharmacognosy, Chemical Profile and Antioxidant Activity of Extracts from Phania matricarioides (Spreng.) Griseb. Collected from Different Localities in Cuba. Plants 7. 10.3390/PLANTS7040110 10.3390/plants7040110 PMC6313911 30558108

[b0070] Harish C.C., Safiullah A., Shenbagaraman R., Premaraj V.S., Venkatraman S.R., Balaji V.A. (2010). Antibacterial activity of Vicoa indica and Tridax procumbens against Multi-Drug Resistant (MDR) clinical isolates. Sci. Trans. Environ. Technovation.

[b0075] Helal, I.M., El-Bessoumy, A., Al-Bataineh, E., Joseph, M.R.P., Rajagopalan, P., Chandramoorthy, H.C., Ben Hadj Ahmed, S., 2019. Antimicrobial Efficiency of Essential Oils from Traditional Medicinal Plants of Asir Region, Saudi Arabia, over Drug Resistant Isolates. Biomed Res. Int. 2019, 8928306. 10.1155/2019/8928306 10.1155/2019/8928306 PMC6354153 30792999

[b0080] Jakubiec-Krzesniak K., Rajnisz-Mateusiak A., Guspiel A., Ziemska J., Solecka J. (2018). Secondary metabolites of actinomycetes and their antibacterial, antifungal and antiviral properties. Polish J. Microbiol..

[b0085] Kondhare D., Lade H. (2017). Phytochemical profile, aldose reductase inhibitory, and antioxidant activities of Indian traditional medicinal Coccinia grandis (L.) fruit extract. 3. Biotech.

[b0090] Manavathu E.K., Alangaden G.J., Lerner S.A. (1996). A comparative study of the broth micro- and macro-dilution techniques for the determination of the in vitro susceptibility of Aspergillus fumigatus. Can. J. Microbiol..

[b0095] Mukherjee K., Ghosh N.C., Datta T. (1972). Coccinia indica Linn. as potential hypoglycaemic agent. Indian J. Exp. Biol..

[b0100] Niazi J., Singh P., Bansal Y., Goel R.K. (2009). Anti-inflammatory, analgesic and antipyretic activity of aqueous extract of fresh leaves of Coccinia indica. Inflammopharmacology.

[b0105] Noorulla K.M., Prabahar A.E., Biswas A., Chavala A., Kumar R., Karthikeyan R. (2009). Anthelmitic Potential of Roots of Coccinia Indica Wigh & Arn. Pharmacologyonline.

[b0110] Oboh I.E., Obasuyi O., Akerele J.O. (2008). Phytochemical and comparative antibacterial studies on the crude ethanol and aqueous extracts of the leaves of Lecaniodiscus cupanoides Planch (Sapindaceae). Acta Pol. Pharm..

[b0115] Raje V.N., Yadav A.V., Shelar P.A. (2013). Coccinia indica- A phytopharmacological review - ProQuest. Res. J. Pharmacogn. Phytochem. Raipur.

[b0120] Ramachandran, A., Prasath, R., Anand, A., 2014. The medical uses of Coccinia grandis L. Voigt: A review | INTERNATIONAL JOURNAL OF PHARMACOGNOSY [WWW Document]. 1. URL https://ijpjournal.com/bft-article/the-medical-uses-of-coccinia-grandis-l-voigt-a-review/ (accessed 10.14.21).

[b0125] Sakharkar P., Chauhan B. (2017). Antibacterial, antioxidant and cell proliferative properties of Coccinia grandis fruits. Avicenna J. phytomedicine.

[b0130] Shaheen S., Bolla K., Vasu K., Charya M. (2011). Antimicrobial activity of the fruit extracts of *Coccinia indica*. African J. Biotechnol..

[b0135] Shifali T., Hemlata K., Gitika C. (2021). Coccinia indica (Kundru): A magical herb with antidiabetic potential. Int. J. Ayurveda Pharma Res..

[b0140] Silva E., Fernandes S., Bacelar E., Sampaio A. (2016). Antimicrobial activity of aqueous, ethanolic and methanolic leaf extracts from Acacia spp. and Eucalyptus nicholii. African J. Tradit. Complement. Altern. Med. AJTCAM.

[b0145] Simpkin V.L., Renwick M.J., Kelly R., Mossialos E. (2017). Incentivising innovation in antibiotic drug discovery and development: progress, challenges and next steps. J Antibiot.

[b0150] Venkateswaran S., Pari L. (2003). Effect of Coccinia indica leaf extract on plasma antioxidants in streptozotocin- induced experimental diabetes in rats. Phytother. Res..

[b0155] Yi T., Lo H., Zhao Z., Yu Z., Yang Z., Chen H. (2012). Comparison of the chemical composition and pharmacological effects of the aqueous and ethanolic extracts from a Tibetan “Snow Lotus” (Saussurea laniceps) herb. Molecules.

[b0160] Yuan H., Ma Q., Ye L.i., Piao G. (2016). The traditional medicine and modern medicine from natural products. Molecules.

